# *GRIN2A* null variants confer a high risk for early-onset schizophrenia and other mental disorders and potentially enable precision therapy

**DOI:** 10.1038/s41380-025-03279-4

**Published:** 2025-10-14

**Authors:** Johannes R. Lemke, Andrea Eoli, Ilona Krey, Bernt Popp, Vincent Strehlow, Dirk A. Wittekind, Anna-Leena Vuorinen, Hesham M. Aldhalaan, Sarah Baer, Anne de Saint Martin, Trine B. Hammer, Isabella Herman, Frauke Hornemann, Trine Ingebrigtsen, Damien Lederer, Gaetan Lesca, Dana Marafie, Mikael Mathot, Jill A. Rosenfeld, Rikke S. Møller, Helenius J. Schelhaas, Chelsey Stillman, Alessandro Orsini, Anup D. Patel, Juliette Piard, Pierangelo Veggiotti, Danique R. M. Vlaskamp, Sarah Weckhuysen, Stephen F. Traynelis, Tim A. Benke, Henrike O. Heyne, Steffen Syrbe

**Affiliations:** 1 Institute of Human Genetics, University of Leipzig Medical Center, Leipzig, Germany; 2 Center for Rare Diseases, University of Leipzig Medical Center, Leipzig, Germany; 3 Hasso Plattner Institute, Digital Engineering Faculty, University of Potsdam, Potsdam, Germany; 4 Windreich Department of Artificial Intelligence and Human Health, Icahn School of Medicine at Mount Sinai, New York, NY USA; 5 Department of Psychiatry and Psychotherapy, University of Leipzig Medical Center, Leipzig, Germany; 6 Institue for Laboratory Medicine, Clinical Chemistry and Molecular Diagnostics, University of Leipzig Medical Center, Leipzig, Germany; 7 Institute for Molecular Medicine Finland, University of Helsinki, Helsinki, Finland; 8 Faculty of Social Sciences, Unit of Health Sciences, Tampere University, Tampere, Finland; 9 Department of Neuroscience Centre, King Faisal Specialist Hospital and Research Centre, Riyadh, Kingdom of Saudi Arabia; 10 Department of Pediatric Neurology, University Hospitals of Strasbourg, Strasbourg, France; 11 Department of Clinical Genetics, Copenhagen University Hospital, Rigshospitalet, Copenhagen, Denmark; 12 Department of Epilepsy Genetics and Personalized Medicine, The Danish Epilepsy Centre, Dianalund, Denmark; 13 Department of Molecular and Human Genetics, Baylor College of Medicine, Houston, TX USA; 14 Department of Pediatrics, Klinikum Chemnitz, Chemnitz, Germany; 15 National Centre for Epilepsy, Oslo University Hospital, Oslo, Norway; 16 Centre de Génétique Humaine, Institut de Pathologie et Génétique (IPG), Charleroi (Gosselies), Belgium; 17 Department of Medical Genetics, University Hospital of Lyon, Claude Bernard University Lyon 1, Lyon, France; 18 Department of Pediatrics, Faculty of Medicine, Kuwait University, Safat, Kuwait; 19 Neuropediatric Unit, CHU UCL-Namur, Namur, Belgium; 20 Baylor Genetics Laboratory, Houston, TX USA; 21 Department of Regional Health Research, University of Southern Denmark, Odense, Denmark; 22 Department of Neurology, Stichting Epilepsie Instellingen Nederland, SEIN, Zwolle, The Netherlands; 23 Section of Child Neurology, Department of Pediatrics, University of Colorado, Aurora, CO USA; 24 Section of Pediatric Neurology, Division of Pediatrics, Department of Clinical and Experimental Medicine, University of Pisa, Pisa, Italy; 25 Division of Pediatric Neurology, Nationwide Children’s Hospital, Department of Pediatrics, The Ohio State College of Medicine, Columbus, OH USA; 26 Centre de Génétique Humaine, Centre Hospitalier Universitaire, Université de Franche-Comté, Besançon, France; 27 UMR 1231 GAD, Inserm, Université de Bourgogne, Dijon, France; 28 Neuroscience Research Center, Department of Biomedical and Clinical Sciences, University of Milan, Milan, Italy; 29 Pediatric Neurology Unit, Buzzi Children’s Hospital, Milan, Italy; 30 Department of Genetics and Department of Neurology, University Medical Center Groningen, Groningen, The Netherlands; 31 VIB-Center for Molecular Neurology, VIB, Antwerp, Belgium; 32 Department of Neurology, University Hospital Antwerp, Antwerp, Belgium; 33 Faculty of Medicine and Health Science, University of Antwerp, Antwerp, Belgium; 34 Department of Pharmacology and Chemical Biology, Emory University School of Medicine, Atlanta, GA USA; 35 Center for the Functional Evaluation of Rare Variants (CFERV), Emory University School of Medicine, Atlanta, GA USA; 36 Finnish Institute for Molecular Medicine (FIMM), University of Helsinki, Helsinki, Finland; 37 Heidelberg University, Medical Faculty of Heidelberg, Center for Child and Adolescent Medicine, Clinic 1, Division of Pediatric Epileptology, Heidelberg, Germany

**Keywords:** Schizophrenia, Genetics

## Abstract

Rare genetic factors have been shown to substantially contribute to mental illness, but so far, no precision treatments for mental disorders have been described. It was recently identified that rare variants in *GRIN2A* encoding the GluN2A subunit of the N-methyl-D-aspartate receptor (NMDAR) confer a substantial risk for schizophrenia. To determine the prevalence of mental disorders among individuals with *GRIN2A*-related disorders, we enquired the presence of psychiatric symptoms in 235 individuals with pathogenic variants in *GRIN2A* who had previously enrolled in our global GRIN registry. We identified null variants in *GRIN2A* (*GRIN2A*_null_) to be significantly associated with a broad spectrum of mental disorders including schizophrenia compared to a longitudinal population cohort (FinRegistry) as well as missense variants (*GRIN2A*_missense_). In our cohort, *GRIN2A*_null_-related mental disorders manifest in early childhood or adolescence, which is substantially earlier than the average adult onset in the general population. In 68% of co-incident epilepsy and mental disorder, mental disorders start after epilepsy offset and the age of epilepsy offset correlated with mental disorder onset. *GRIN2A*_null_-related phenotypes appear to occasionally even manifest as isolated mental disorder, i.e. as schizophrenia or mood disorder without further *GRIN2A*-specific symptoms, such as intellectual disability and/or epilepsy. As L-serine is known to mediate co-agonistic effects on the NMDAR, we applied it to four individuals with *GRIN2A*_null_-related mental disorders, all of whom experienced improvements of their neuropsychiatric phenotype. *GRIN2A*_null_ appears to be the first monogenic cause of early-onset and even isolated mental disorders, such as early-onset schizophrenia. Genetic testing should be considered in the diagnostic work-up of affected individuals to improve diagnosis and potentially offer personalized treatment as increasing brain concentrations of NMDAR co-agonists appears to be a promising precision treatment approach successfully targeting deficient glutamatergic signaling in individuals with mental disorders, i.e. due to *GRIN2A*_null_.

## Introduction

Mental disorders pose a substantial burden to the affected individual and society, but treatment advances have been limited by our incomplete understanding of their molecular etiology. [1] According to the World Health Organization, in 2019 one in every eight people was living with a mental disorder worldwide, such as anxiety, depression, eating disorder or schizophrenia (https://www.who.int/news-room/fact-sheets/detail/mental-disorders).

Mental disorders have complex causes with a substantial genetic contribution. Having an affected close family member has been considered one of the highest known risk factors. [2] Previous studies considered mental disorders to be highly polygenic [3] and identified thousands of common genetic variants, each contributing a small risk increment. [4–6] A shared pathophysiological etiology and the implication of similar biological pathways have been suggested due to a high degree of shared genetic effects across different types of mental disorders [7, 8] as well as overlapping clinical presentations.

Among mental disorders, schizophrenia was shown to have a particularly high degree of heritability. [4, 9] Rare protein-truncating null variants in numerous genes have been considered to contribute to the heritability of schizophrenia and other mental disorders. [10, 11] These typically cause a much larger increase in disease risk than individual common variants, but are present in a smaller proportion of affected individuals. [12] The reduced reproductive rate of individuals with schizophrenia reduces the transmission rate of any large heritable risk factors in the population. [13] Risk variants with strong effects thus usually arise de novo, not being present in either of the parents. Few genetic factors conferring considerable risk increments for schizophrenia have been identified – in particular copy number variants (CNV) involving large chromosomal segments usually covering multiple genes. [14–16] The majority of individuals with mental disorders due to such de novo CNV display a recognizable syndromic phenotype comprising intellectual disability (ID) and/or other syndrome-associated features, including dysmorphism and congenital malformations. [17] The most prevalent example is 22q11.2 deletion syndrome, where up to 24% of affected adults are known to develop schizophrenia [18], and vice versa, up to 1% of individuals with schizophrenia are described to have a 22q11.2 deletion. [19–21] Another CNV on 16p13.2 containing *GRIN2A*, a gene encoding the GluN2A subunit of the N-methyl-D-aspartate receptor (NMDAR) and previously associated with various epilepsy phenotypes [22, 23], is one of the very few loci that is recurrently associated with various mental disorders, i.e. bipolar disorders [24] and schizophrenia [25]. Still, CNV analysis or even exome- or genome-sequencing is not part of the recommended routine diagnostic work-up in individuals with mental disorders (https://ispg.net/genetic-testing-statement/).

While rare de novo variants affecting individual genes have been identified to contribute to schizophrenia globally [26, 27], only a single gene, *SETD1A*, has been robustly associated with schizophrenia, previously. [28] This is, however, in the context of a syndromic and severe neurodevelopmental disorder as well as an adult-onset psychiatric condition. Isolated mental disorders, particularly non-syndromic psychiatric phenotypes, have not yet been associated with alterations of a single gene conferring a large risk with a Mendelian inheritance pattern. [29, 30]

A recent exome sequencing study identified rare coding variants in 10 genes conferring substantial risk for schizophrenia, including protein-truncating null variants in *GRIN2A* (*GRIN2A*_null_; overall p-value 7×10^−7^, Odds Ratio = 24.1). [16] This is in agreement with our previous report of three individuals with *GRIN2A*_null_ from our GRIN registry (https://grin-portal.broadinstitute.org/) with either psychotic disorders (n = 2) or anxiety disorder (n = 1). [23] Our registry comprises 235 individuals with *GRIN2A*-related disorders that were not ascertained for mental disorders, and thus provides a unique opportunity to systematically investigate the incidence of mental disorders across the lifetime and its association with other symptoms of *GRIN2A*-related disorders, such as epilepsy. This is of particular relevance as the presence of epilepsy alone significantly increases the prevalence of mental illness with respective prevalence rates of up to ~35% for mood disorders, ~25% for anxiety disorders, ~5% for psychotic disorders (and 1.7% for schizophrenia in particular). [31–33]

The orally available non-essential amino acid L-serine mediates co-agonistic effects on the NMDAR in neurons via its enantiomer D-serine. [34] We recently reported on improved behavior, electroencephalography (EEG) and seizure frequency in individuals with *GRIN2A*_null_ treated with L-serine. [35] We therefore also collected retrospective observational data on treatment responses to L-serine in this cohort of individuals with *GRIN2A*_null_-related mental disorders.

## Methods

### Screening cohort

We contacted the treating physicians of all 235 individuals with *GRIN2A*-related disorders who had previously been in contact with us or our registry of GRIN-related disorders (http://grin-portal.broadinstitute.org/). All 235 individuals (Supplementary Table 1) carried (likely) pathogenic variants according to the American College of Medical Genetics and Genomics classification guidelines. [36] G*RIN2A* variants of uncertain significance were disregarded in the present genotype-phenotype association study. The vast majority of study individuals already participated in our previous *GRIN2A* genotype-phenotype association study, which did not yet include a systematic evaluation of psychiatric symptoms. [23] According to the Diagnostic and Statistical Manual of Mental Disorders and the International Classification of Diseases, mental disorders comprise anxiety, mood, psychotic, personality and eating disorders as well as numerous conditions and sub-entities that share fluent borders to various behavioral, neurologic and/or developmental disorders. [37]

Consequently, we requested information/confirmation for all 235 individuals addressing:

- i) patient age (at last consultation)
- ii) sex
- iii) presence or absence of any mental disorder (i.e. anxiety, mood, psychotic, personality and/or eating disorders or other). [37]

We received a reply for 196 of 235 (83.4%) individuals, comprising 78 males, 73 females, and 45 cases of unspecified sex. Among these 196 individuals, age was known in 151 individuals (mean 18.3 years, range 1–62 years). In 121 out of the 196 (61.7%) cases on which we received a reply, there was a clear answer on the presence (n = 25/121; 20.7%) or absence (n = 96/121; 79.3%) of mental disorders. Among the remainder (n = 75), this aspect could not be clarified (e.g., due to lack of respective data in the records and/or loss of contact with the affected individual), and these individuals were thus excluded from further analyses.

For all individuals on which we received confirmation of one or more mental disorders, we requested more detailed retrospective phenotyping with particular focus on psychiatric aspects (i.e. psychiatric symptoms and/or diagnoses, age of onset, treatment, persistence of symptoms or age of offset) according to a standardized questionnaire (Supplementary Table 2).

### Statistical analyses, data processing and code availability

Statistics were done using R version 4.2.1 (2022-06-23). Survival analyses and Cox Proportional Hazard (Cox-PH) models were carried out using the R packages survminer (https://CRAN.R-project.org/package=survminer) and survival (https://CRAN.R-project.org/package=survival). The complete reproducible code is available on GitLab (https://gitlab.hpi.de/andrea.eoli/grin2a-and-mental-disorders) upon reasonable request. When analyzing individual mental disorders, we counted as cases each carrier of a *GRIN2A* pathogenic variant that had at least one mental disorder. Carriers with multiple diagnoses were counted in each category and with the respective disease onset ages when available. In a few cases, the treating physicians answered with a text instead of numbers (e.g., “as a child”, “childhood”, or “teenager”) in the fields “epilepsy onset”, “epilepsy offset”, or “psychiatric onset”. In those cases, we used the respective medians as approximations. Additionally, a few individuals were diagnosed with a psychiatric disorder, but the related age of onset was missing. We used the “age at last examination” as an approximation for those individuals when it was lower than the highest “psychiatric onset” available.

### Control cohort

The incidence case count of mental disorders was extracted from the FinRegistry Data, which is a nationwide cohort study including all Finnish citizens alive on Jan 1^st^ 2010 as well as their close relatives, and linking their health and social data from 19 registers. [38] FinRegistry data are mapped to more than 3000 clinical endpoints defined by leveraging registers and clinical expertise (disease distributions available at https://risteys.finregistry.fi). For the current study, we included individuals alive on Jan 1^st^ 2010 and their inpatient and outpatient data from 1998 onwards. Using the clinical endpoint definitions, we analyzed the following disease categories: anxiety disorder, mood disorders, and psychotic disorders (see Supplementary Table 3 for exact endpoint definitions). For the analyses we included the occurrence of the first diagnosis only. The follow-up time was defined from the date of birth until the date of symptoms, or emigration, or death, of the end of the follow-up 31.12.2019, whichever came first. We included as cases individuals who received a diagnosis between 1 and 80 years of age to match the same age range with our cohort. These data were used to illustrate the cumulative incidence of the three disorders. Additionally, using the sex-specific lifetables, we generated synthetic data for a cohort comprising 100,000 individuals. Subsequently, we utilized this synthetic dataset to conduct a comparative analysis of disease incidences between control subjects and the *GRIN2A* population using Cox Proportional Hazards regression. For each mental disorder in *GRIN2A*_null_ carriers, the regression was limited to the age bins in which the model assumptions were met: 0–12 years old for anxiety disorders, 0–11 years old for mood disorders, 0–12 years old for psychotic disorders.

### Prevalence of psychiatric disorders in individuals with epilepsy

To compare the prevalence of psychiatric disorders between the subset of our *GRIN2A* cohort with epilepsy and published epilepsy cohorts, we conducted one-sided Fisher’s exact tests on the reported counts of affected individuals. Analyses were performed separately for *GRIN2A*_null_ and *GRIN2A*_missense_, using data extracted from Reilly et al. (age range: 5–15 years; N = 85) [39] and Aaberg et al. (age range: 0–17 years; N = 6635) [40] as control groups, respectively. Nominal p-values are reported; for reference, the Bonferroni-corrected significance threshold for four tests is p < 0.0125. (Supplementary Table 5) Data for both *GRIN2A*_null_ and *GRIN2A*_missense_ has been collected identically and is thus subject to identical potential biases. Moreover, both cohorts comprise individuals with similar prevalence, spectrum and severity of epilepsy and even accompanying intellectual disability and age range. In particular, our previous study on *GRIN2A*-related disorders revealed that the prevalence of epilepsy in individuals with *GRIN2A*_missense_ (79/90; 87.8%) is almost identical to that seen in individuals with *GRIN2A*_null_ (113/129; 87.6%). [23]

### Precision therapy with L-serine in *GRIN2A*_null_ carriers

We also collected retrospective observational data on four individuals of our cohort that had been treated with L-serine, two of which had been published previously. [35] (Table 1)

**Table 1:** Precision therapy with L-serine in *GRIN2A*_null_ carriers.

ID	r174 (#9 from Krey et al. 2022) [35]	r171 (#10 from Krey et al. 2022) [35]	r236 (novel)	r228 (novel)
Variant	GRIN2A c.176_179dupAGGC p.(Ala61Glyfs*78)	GRIN2A c.(2168 + 1_2169-1)_(4395_?)del) p.(?)	GRIN2A c.280_283delCGCA p.(Arg94Serfs*15)	GRIN2A c.785_786insTC p.(Gly263Leufs*31)
Sex	female	male	female	male
Age at last follow-up	13 years	15 years	17 years	7 years
Mental disorder	Hallucinations	Anxiety and hallucinations	Psychotic disorder and paranoia	Depression and anxiety
Other symptoms	CSWS, no ID, aggressive behavior	Landau-Kleffner syndrome, focal epilepsy, mild ID, hyperactive behavior, oral dyspraxia	Focal epilepsy, mild ID, dysarthria, aphasia, anomic behavior	Focal epilepsy, epileptic spasms, mild ID, aggressive behavior
Age at start of L-serine treatment	12 years	14 years	16 years	unknown
Duration of L-serine treatment	7 months; 4 months stop; >12 months	>12 months	12 months	unknown
L-serine dose (mg/kg/d)	100; 500	125	500	500
L-serine treatment effect	Stop of hallucinations, improved behavior	Improved behavior	Remission of paranoid symptoms	Reduced seizure frequency

Clinical details and therapy response of individuals with *GRIN2A*_null_ and mental disorders treated with L-serine.

*CSWS* continuous spikes and waves during slow-wave sleep, *ID* intellectual disability.

## Results

### Screening cohort

We obtained data on the presence or absence of mental disorders for 121 individuals carrying pathogenic variants in *GRIN2A*, comprising 25 individuals with mental disorders and 96 without. An additional 114 individuals were excluded from further analyses due to unclear mental health status, which could be due to missing data or lost contact. Among the 25 individuals diagnosed with mental disorders, at least 18 were diagnosed by a psychiatrist or a neurologist with psychiatric expertise and/or receiving psycho-pharmacologic treatment and/or psychotherapy. Thus, these clinical diagnoses were considered valid for our *GRIN2A* study population. This cohort consisted of 102 index cases and 19 relatives. To minimize ascertainment-related biases, we analyzed these groups separately. Summary statistics are presented in Table 2. Individuals with mental disorders were significantly older than those without, in the full cohort (p = 0.003, one-sided unpaired Wilcoxon test) and when considering only index cases (p = 0.007, Table 2, Fig.S1).

**Table 2:** Cohort’s summary statistics.

	Cohort size	Mean age	Median age [min-max]
**All cases**	**121**	**18.3**	**13** [1–62]
-MD	25	24.5	16 [10–55]
-Non-MD	96	16.8	12 [1–62]
**Index cases**	**102**	**14.1**	**12** [1–57]
-MD	19	18	15 [10–48]
-Non-MD	83	13.3	11 [1–57]
**Family members**	**19**	**41.1**	**40** [25–62]
-MD	6	45	45.5 [36–55]
-Non-MD	13	39.3	40 [25–62]

Summary statistics for the entire *GRIN2A* study cohort and its two sub-cohorts (“Index cases” and “Family members”). Participants are categorized based on the presence or absence of mental disorders (‘MD’ and ‘Non-MD’). The age is expressed in years and the median age includes the minimum-maximum range.

In the cohort of 121 individuals, 84 (69.4%) carried *GRIN2A*_null_ and 37 (30.6%) *GRIN2A*_missense_. Among the 25 individuals with mental disorders, the underlying pathogenic or likely pathogenic *GRIN2A* variants comprised 23 *GRIN2A*_null_ and two *GRIN2A*_missense_. Individuals with *GRIN2A*_null_ had thus a significantly higher probability of mental disorders compared with carriers of *GRIN2A*_missense_ (23/84 vs 2/37; Fisher’s exact test p = 0.006; OR = 6.5, CI_95%_ = [1.4, 60.4]).

Similar results are obtained when applying a logistic regression model to control for age, sex, and whether the individual is a family member of an index case. The model confirms that individuals with *GRIN2A*_null_ variants are significantly associated with an increased risk of mental disorders (p = 0.027, OR = 5.7, CI_95%_ = [1.2, 26.6]). Age, sex and being a family member do not significantly influence the outcome in the current analysis.

### Carriers of *GRIN2A*_null_ have an elevated incidence of childhood-onset mental disorders

Twenty three of 84 *GRIN2A*_null_ carriers had mental disorders, 13 had mood disorders, twelve anxiety disorders, eight psychotic disorders, three personality disorders and one had eating disorder. Twelve *GRIN2A*_null_ carriers had complex psychiatric phenotypes of more than one mental disorder. Two out of 37 individuals with *GRIN2A*_missense_ had isolated mood or isolated anxiety disorder (n = 1, each).

**Fig. 1:**
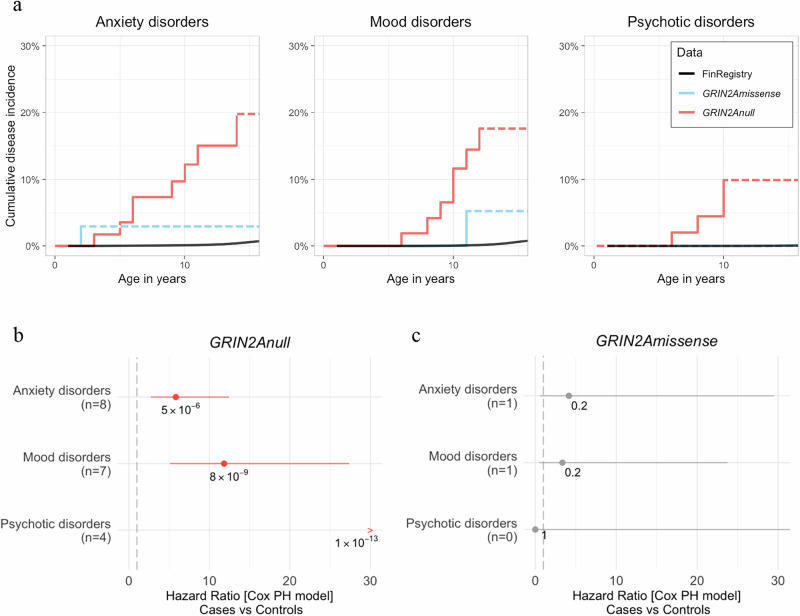
Cumulative incidence of mental disorders and Hazard Ratios for index carriers of pathogenic *GRIN2A* variants carriers. **a** Cumulative disease incidence in individuals between 0 and 15 years old for anxiety disorders, mood disorders, and psychotic disorders, respectively. The black line represents the global disease incidence (FinRegistry data), the light blue line is the disease incidence for carriers of *GRIN2A*_missense_, and the red line is the disease incidence for carriers of *GRIN2A*_null_. The dashed line illustrates the lack of age-of-onset data in older age ranges. **b, c** Hazard Ratios for these three disease groups, for index *GRIN2A*_null_ carriers (**b**) and index *GRIN2A*_*missense*_ carriers (**c**). The number of cases (n) is specified under the disease name. The number of controls (FinRegistry data) for the same age bins is 200 for anxiety disorders, 90 for mood disorders, and 10 for psychotic disorders. The vertical dashed line represents the value at which the hazard is the same in both groups (HR = 1). Statistically significant results (adjusted p < 0.05) are highlighted in red, and the nominal p-value is shown in the figure.

For each *GRIN2A*_*null*_-related mental disorder, we systematically compared cumulative disease incidence of *GRIN2A*_*null*_ carriers versus the average population using the FinRegistry data as a nationwide control cohort, containing longitudinal electronic health records from the whole Finnish population (>5 Million individuals, spanning 21 years of individual-level data). [38] In 65.2% of cases (15 of 23), the onset age was available for at least one mental disorder. To reduce a potential selection bias, we excluded family members from the following analyses, therefore focusing on the *GRIN2A*_null_ index cases, only. For adequate power, we further focused on individual mental disorders in which we had ≥4 observations with available onset data in *GRIN2A*_null_ carriers: anxiety disorders, mood disorders, and psychotic disorders. In all three disease categories, *GRIN2A*_null_ carriers had a significantly higher incidence of mental disorders compared to the control cohort (Fig.1). We restricted the analysis to age bins in which the hazard is proportional, as required by the Cox-PH model. *GRIN2A*_null_ carriers showed elevated lifetime incidence of psychotic disorders (HR = 87, CI_95%_ = [26.7-283.4], p = 1.3e-13, n = 4, age bins = 0-12), mood disorders (HR = 11.8, CI_95%_ = [5.1-27.4], p = 7.9e-9, n = 7, age bins = 0-11), and anxiety disorders (HR = 5.84, CI_95%_ = [2.7-12.4], p = 4.9e-6, n = 8, age bins = 0-12). We did not find a statistically significant difference in the incidence of mental disorders between individuals with pathogenic *GRIN2A*_*missense*_ variants and the control cohort. Furthermore, we checked whether *GRIN2A*_null_ variants would differently affect the incidence of the three mental disorders (Fig.S2) and found no significant difference between the three incidence curves (Log-Rank test p = 0.7, testing both globally and pairwise, adjusting for multiple testing with the Benjamini-Hochberg procedure).

Lastly, we identified six individuals with oligosymptomatic *GRIN2A*_*null*_-related phenotypes, i.e. isolated non-syndromic mental disorder without other *GRIN2A*-specific features, such as ID (absent in 6/6) or epilepsy (absent in 2/6, both of which are adult carriers). Of these six individuals, four are adult carriers and family members of other index cases.

### Age at epilepsy offset correlates with onset of mental disorder in *GRIN2A*_null_ carriers

**Fig. 2:**
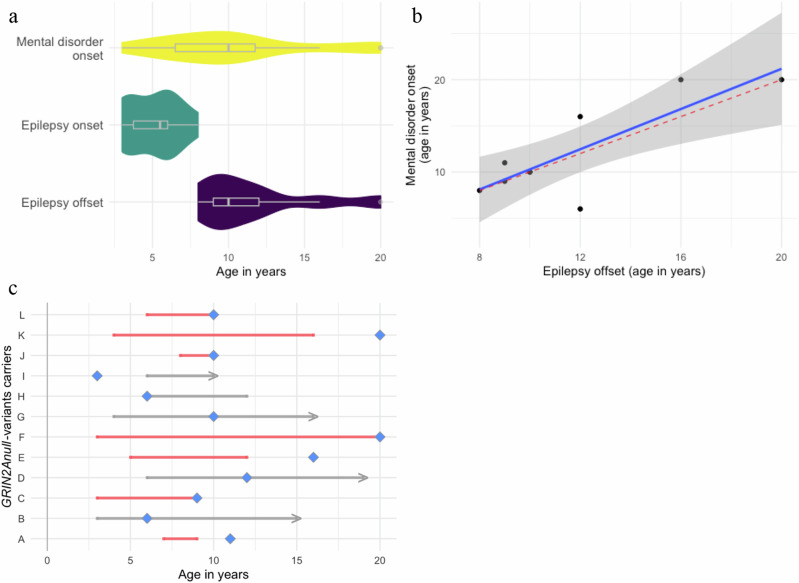
Analysis of age of onset and offset for epilepsy and the age of psychiatric onset. **a** Violin plot with boxplot for individuals where we have information on age of mental disorder onset, epilepsy onset, and epilepsy offset. **b** Scatter plot of the mental disorder onset with the epilepsy offset, in which a linear model has been fitted (var: epilepsy offset, p = 0.007, ß = 1.43, residual SE = 2.92, R^2^ = 0.68); we adjusted the model for sex. The grey band represents the 95% confidence interval, and the red dashed line represents the x = y relationship as comparison. **c** Age timeline of carriers of *GRIN2A*_null_. The age period with a diagnosis of epilepsy is represented by a line, which is an arrow in case of an ongoing epilepsy. The blue diamond represents the onset of mental disorder. The line is colored in red when the age of the psychiatric onset is the same or higher than the age of epilepsy offset.

We aimed to investigate whether the presence of mental disorders within our whole cohort was associated with the presence of epilepsy or intellectual disability. Among *GRIN2A*_null_ carriers with a mental disorder, epilepsy was diagnosed in 82.6% of cases (19 of 23), and epilepsy onset and offset data were available for twelve of these individuals. While we found no significant difference in the incidence of mental disorders between individuals with and without epilepsy (p = 0.63, Log-Rank test; Fig. S3), we identified a correlation between the offset of epilepsy and the onset of a mental disorder (p = 0.007, R^2^ = 0.63, residual SE = 3.15, method: Spearman; Fig. 2b). This correlation is also evident when plotting the age at epilepsy offset and mental disorder onset as a timeline (Fig. 2c). In 58% of cases (7/12), the mental disorder occurred *after* epilepsy offset. Additionally, we examined whether mental disorder incidence in *GRIN2A*_null_ carriers varied based on ID or seizure type and found no statistically significant differences (p = 0.73, for seizure type, p = 0.073 for ID status, Log-Rank test; Fig. S3).

### Precision therapy with L-serine in *GRIN2A*_null_ carriers

Within our overall cohort, four individuals with *GRIN2A*_null_-related mental disorders were treated open label with up to 500 mg/kg/d L-serine over >12 months. [35] A retrospective observational data collection revealed that all four individuals experienced phenotypic improvements. Individual r174 (identical to #9 from Krey et al.) [35] showed improvements of psychotic symptoms and ceasing of hallucinations. Individual r171 (identical to #10 from Krey et al.) [35] showed improvements of behavioral disorder. Two additional and previously unpublished individuals showed similar beneficial treatment responses comprising remission of paranoid symptoms in r236 and a reduction of seizure frequency in r228. (Table 1)

## Discussion

With the currently largest international cohort of individuals with disease-causing *GRIN2A* variants, we identify that *GRIN2A*_null_ variants significantly increase risk for a variety of mental disorders. The average age of onset for *GRIN2A*_null_-related mental disorders was childhood or adolescence. *GRIN2A*_null_ carriers were thus at specifically elevated risk at a much younger age than the global population average. While we replicate the initial association of *GRIN2A*_null_ and schizophrenia of the exome-wide SCHEMA consortium [16], we find that the elevated mental disorder risk of *GRIN2A*_null_ carriers is not restricted to schizophrenia but comprises a much broader spectrum, including most evidently early-onset mood as well as anxiety disorders, which is in contrast to *GRIN2A*_missense_ carriers, where mental disorders have barely been diagnosed.

*GRIN2A* has initially been associated with neurodevelopmental disorders, comprising particularly developmental and epileptic encephalopathy, ID and speech disorders. [22, 23, 41] Our findings indicate that mental disorders are not just one of many features of a complex neurodevelopmental phenotype of *GRIN2A*_null_-related disorder but can be a leading clinical presentation rather than a co-morbidity or concomitant feature of epilepsy. (Fig. 1 and Supplementary Table 5) Given the same prevalence of epilepsy among individuals with *GRIN2A*_null_ and *GRIN2A*_missense_, we conclude that the presence of mental disorders among individuals with *GRIN2A*_null_ cannot easily considered to be a consequence or concomitant symptom of the diagnosis of epilepsy. In about half the individuals affected by epilepsy as well as mental disorders, the mental disorder started after the offset of epilepsy. However, it currently remains unclear whether the correlation between offset of epilepsy and onset of mental disorders has a biological cause. The fact that *GRIN2A*_null_-related disorders can present as an oligosymptomatic phenotype, i.e. as an isolated non-syndromic mental disorder without ID (n = 6) and even also without epilepsy (n = 2 of these 6), uncovers not only a completely novel phenotypic spectrum within this monogenetic entity but challenges our perception of isolated mental disorder as a complex and polygenic disease [3]. The International Society of Psychiatric Genetics does not currently recommend genetic testing in the diagnostic work-up of isolated mental diseases (https://ispg.net/genetic-testing-statement/), which is in contrast to e.g. epilepsy disorders. [42, 43] Due to the ascertainment bias that prioritizes individuals with severe neurodevelopmental presentations for genetic testing, the proportion of *GRIN2A*_null_ carriers with isolated mental disorders is likely even higher than in our cohort.

A potential association of *GRIN2A*_null_ with monogenic and even isolated mental disorders may thus require a re-evaluation of the role of genetic testing within the diagnostic work-up of individuals with mental disorders, particularly in case of early onset. We suggest to consider similar recommendations for genetic testing as established in other neurodevelopmental disorders, such ID and/or early-onset epilepsy [42, 43].

As a limitation of our study, the incidence of mental disorders was ascertained with targeted questionnaires, which differs from the FinRegistry control data. The diagnosis of mental symptoms followed standards in the respective institutions, and data were collected retrospectively. Given that 18 out of 25 diagnoses were made by experienced clinicians and/or were accompanied by specific psychiatric treatments, we consider these diagnoses valid. In addition, our control cohort comes from well-powered nationwide longitudinal Finnish registry data counting any mental disorder diagnosis irrespective of primary care or hospital setting. We thus expect to be conservative in not underestimating the presence of mental disorders in our control cohort.

Independently, we observe this association solely for *GRIN2A*_null_ but not *GRIN2A*_missense_, which is in line with the association of *GRIN2A*_null_ previously observed in a large case control exome study. [16] Due to a primary ascertainment through genetic testing for childhood-onset neurodevelopmental disorders, our observations are biased to a younger age range and more severe phenotypes. A benefit of this ascertainment is, however, an unbiased estimate of mental disorder incidence for that cohort. The incidence of isolated *GRIN2A*_null_-related mental disorders without neurodevelopmental disorders and/or epilepsy is thus likely an underestimate in our data.

In addition to diagnostic certainty, establishing a genetic diagnosis of *GRIN2A*-related disorders can have therapeutic consequences. We previously reported evidence for a therapeutic benefit of L-serine in individuals with *GRIN2A*_null_-related disorders. [35] These benefits predominantly affected behavior, but also developmental and epilepsy-related features. Strikingly, all four individuals in our cohort of *GRIN2A*_null_-related mental disorders who received L-serine experienced phenotypic improvements, such as ceasing of hallucinations, remission of paranoid symptoms or improvement of behavioral disorder (Table 1), being in line with a recent non-randomized clinical trial on L-serine in individuals with *GRIN*-related disorders. [44] As the collection of observational data was performed retrospectively in our study, a prospective randomized and double-blinded placebo-controlled clinical trial would be needed to definitely confirm our observation.

Treatment with L-serine specifically targets the glutamatergic system. The naturally occurring amino acid L-serine is known to be taken up into neurons and converted to its enantiomer D-serine, which not only shares structural similarities with glycine but in fact competes for binding at the glycine modulatory site and is functionally an even more potent activator of synaptic NMDAR than glycine itself. [45, 46] By binding to the GluN1 subunit, D-serine serves as an endogenous co-agonist of the NMDAR. [34] Interestingly, glycine and D-serine have lower potency at GluN2A-containing than other GluN2-containing NMDAR [47], increasing the likelihood that GluN2A-containing receptors have the lowest occupancy of the glycine site at rest. This suggests L-serine-induced increases in D-serine would have a disproportionately larger effect on GluN2A-containing NMDAR, which might partly compensate for reduction in GluN2A expression. D-serine has long been implicated in modulating fear conditioning and anxiety disorders. [34] In rodents, its application mediates antidepressant-like effects [48], is considered a potential treatment for schizophrenia [49], and restores trace fear conditioning impairment in mice lacking serine racemase. [49] Concerning humans, D-cycloserine (another partial agonist of the NMDAR) enhances fear extinction in acrophobia [50] and is modestly effective in treating anxiety disorders. [51] Vice versa, NMDAR channel blockers such as ketamine and phencyclidine were shown to induce psychotic and cognitive symptoms in healthy individuals. [52, 53] While recent studies further support a possible role of NMDAR augmentation in treating e.g. schizophrenia, other meta-analyses failed to demonstrate efficacy. [54] However, the general lack of beneficial effects of D-serine or D-cycloserine in several studies might to some extent also be explained by heterogenous study cohorts and age-dependent benefits. [55] More recent studies implementing different dosing regimens and trial designs again largely support benefit from treatment with D-serine in schizophrenia and other neuropsychiatric disorders [56] and even suggest impaired glutamatergic synaptic function in schizophrenia. [57, 58]

Among the top 10 genes conferring a substantial risk for schizophrenia, the SCHEMA consortium revealed two components of different glutamate receptors, i.e. *GRIN2A* encoding the GluN2A subunit of the NMDAR as well as *GRIA3* encoding the GluR3 subunit of the α-amino-3-hydroxy-5-methyl-4-isoxazolepropionic acid receptor (AMPAR). [16]

A recent study even reported evidence that EEG phenotypes of *Grin2a* mutant mice show a variety of abnormal features that overlap considerably with human schizophrenia patients, reflecting systems-level changes caused by *Grin2a* deficiency. [59] These heterozygous *Grin2a* +/- mice also show a delay in the maturation of parvalbumin interneurons in the hippocampus, which may alter circuit function. [60] All this evidence suggests a potential beneficial effect of activating the NMDAR in various mental disorders.

Strikingly, our data suggests a significant contribution of *GRIN2A*_null_ (but not of *GRIN2A*_missense_) to the development of mental disorders. Thus, an altered assembly and consequently a reduced density of the NMDAR in the cell membrane due to a deficient GluN2A subunit expression due to *GRIN2A*_null_ [23] appears to have a stronger effect on mental health than an altered electrophysiologic receptor function due to *GRIN2A*_missense_ i.e. a loss or a gain of function of the NMDAR. This poses the question of a possible involvement of the yet poorly understood intracellular signaling of the C-terminal domain of GluN2A. C-terminal signaling may still be qualitatively and quantitatively intact in a receptor with altered electrophysiologic properties due to pathogenic *GRIN2A*_missense_. However, it may be quantitatively (not necessarily qualitatively) deficient in a *GRIN2A*_null_ situation, reducing the overall NMDAR density.

The pronounced heterogeneity of mental disorders has so far largely overshadowed the identification of monogenic entities, despite the detection of rare de novo variants particularly in individuals with schizophrenia [26, 27] as well as the association of adult-onset schizophrenia to *SETD1A* in the context of a severe and syndromic neurodevelopmental disorder. [28–30] Thus, our findings establish *GRIN2A*_null_ as the first monogenic predisposition to a broad spectrum of early-onset mental disorders, including early-onset schizophrenia. *GRIN2A*_null_-related phenotypes frequently but not necessarily comprise ID as well as a usually self-limiting childhood epilepsy [60] but may also present as an isolated mental disorder. The observed involvement of dysfunctional subunits of the NMDAR as well as of the AMPAR [16] suggests deficient glutamatergic signaling to substantially contribute to the development of schizophrenia and potentially other mental disorders. This coincides with treatment responses to L-serine as a precision medicine approach enhancing NMDAR-related glutamatergic signaling in *GRIN2A*_null_ carriers, which positively influenced in particular mental and behavioral disorders of several individuals. It remains open and should be subject to clinical trials whether such beneficial treatment responses stay limited to *GRIN2A*_null_-related mental disorders or might be observed even beyond. Lastly, monogenic alterations of this potentially targetable signaling cascade also suggest a potential benefit of genetic testing within the diagnostic work-up of individuals with early-onset mental disorders.

## Supplementary information

Supplementary Information

Supplementary Table 1

Supplementary Table 2

Supplementary Table 3

Supplementary Table 4

Supplementary Table 5
