# Correction: *GRIN2A* null variants confer a high risk for early-onset schizophrenia and other mental disorders and potentially enable precision therapy

**DOI:** 10.1038/s41380-025-03442-x

**Published:** 2026-01-07

**Authors:** Johannes R. Lemke, Andrea Eoli, Ilona Krey, Bernt Popp, Vincent Strehlow, Dirk A. Wittekind, Anna-Leena Vuorinen, Hesham M. Aldhalaan, Sarah Baer, Anne de Saint Martin, Trine B. Hammer, Isabella Herman, Frauke Hornemann, Trine Ingebrigtsen, Damien Lederer, Gaetan Lesca, Dana Marafie, Mikael Mathot, Jill A. Rosenfeld, Rikke S. Møller, Helenius J. Schelhaas, Chelsey Stillman, Alessandro Orsini, Anup D. Patel, Juliette Piard, Pierangelo Veggiotti, Danique R. M. Vlaskamp, Sarah Weckhuysen, Stephen F. Traynelis, Tim A. Benke, Henrike O. Heyne, Steffen Syrbe

**Affiliations:** 1 Institute of Human Genetics, University of Leipzig Medical Center, Leipzig, Germany; 2 Center for Rare Diseases, University of Leipzig Medical Center, Leipzig, Germany; 3 Hasso Plattner Institute, Digital Engineering Faculty, University of Potsdam, Potsdam, Germany; 4 Windreich Department of Artificial Intelligence and Human Health, Icahn School of Medicine at Mount Sinai, New York, NY USA; 5 Department of Psychiatry and Psychotherapy, University of Leipzig Medical Center, Leipzig, Germany; 6 Institue for Laboratory Medicine, Clinical Chemistry and Molecular Diagnostics, University of Leipzig Medical Center, Leipzig, Germany; 7 Institute for Molecular Medicine Finland, University of Helsinki, Helsinki, Finland; 8 Faculty of Social Sciences, Unit of Health Sciences, Tampere University, Tampere, Finland; 9 Department of Neuroscience Centre, King Faisal Specialist Hospital and Research Centre, Riyadh, Kingdom of Saudi Arabia; 10 Department of Pediatric Neurology, University Hospitals of Strasbourg, Strasbourg, France; 11 Department of Clinical Genetics, Copenhagen University Hospital, Rigshospitalet, Copenhagen, Denmark; 12 Department of Epilepsy Genetics and Personalized Medicine, The Danish Epilepsy Centre, Dianalund, Denmark; 13 Department of Molecular and Human Genetics, Baylor College of Medicine, Houston, TX USA; 14 Department of Pediatrics, Klinikum Chemnitz, Chemnitz, Germany; 15 National Centre for Epilepsy, Oslo University Hospital, Oslo, Norway; 16 Centre de Génétique Humaine, Institut de Pathologie et Génétique (IPG), Charleroi (Gosselies), Belgium; 17 Department of Medical Genetics, University Hospital of Lyon, Claude Bernard University Lyon 1, Lyon, France; 18 Department of Pediatrics, Faculty of Medicine, Kuwait University, Safat, Kuwait; 19 Neuropediatric Unit, CHU UCL-Namur, Namur, Belgium; 20 Baylor Genetics Laboratory, Houston, TX USA; 21 Department of Regional Health Research, University of Southern Denmark, Odense, Denmark; 22 Department of Neurology, Stichting Epilepsie Instellingen Nederland, SEIN, Zwolle, The Netherlands; 23 Section of Child Neurology, Department of Pediatrics, University of Colorado, Aurora, CO USA; 24 Section of Pediatric Neurology, Division of Pediatrics, Department of Clinical and Experimental Medicine, University of Pisa, Pisa, Italy; 25 Division of Pediatric Neurology, Nationwide Children’s Hospital, Department of Pediatrics, The Ohio State College of Medicine, Columbus, OH USA; 26 Centre de Génétique Humaine, Centre Hospitalier Universitaire, Université de Franche-Comté, Besançon, France; 27 UMR 1231 GAD, Inserm, Université de Bourgogne, Dijon, France; 28 Neuroscience Research Center, Department of Biomedical and Clinical Sciences, University of Milan, Milan, Italy; 29 Pediatric Neurology Unit, Buzzi Children’s Hospital, Milan, Italy; 30 Department of Genetics and Department of Neurology, University Medical Center Groningen, Groningen, The Netherlands; 31 VIB-Center for Molecular Neurology, VIB, Antwerp, Belgium; 32 Department of Neurology, University Hospital Antwerp, Antwerp, Belgium; 33 Faculty of Medicine and Health Science, University of Antwerp, Antwerp, Belgium; 34 Department of Pharmacology and Chemical Biology, Emory University School of Medicine, Atlanta, GA USA; 35 Center for the Functional Evaluation of Rare Variants (CFERV), Emory University School of Medicine, Atlanta, GA USA; 36 Finnish Institute for Molecular Medicine (FIMM), University of Helsinki, Helsinki, Finland; 37 Heidelberg University, Medical Faculty of Heidelberg, Center for Child and Adolescent Medicine, Clinic 1, Division of Pediatric Epileptology, Heidelberg, Germany

**Keywords:** Schizophrenia, Genetics

Correction to: *Molecular Psychiatry* 10.1038/s41380-025-03279-4, published online 14 October 2025

An error was identified in the Methods section of the original article. The last words in the last line of the section Control Cohort incorrectly referred to “psychiatric disorders” at the end of the paragraph. This was a mistake; the correct term is “psychotic disorders.” The original article has been corrected.

