# Supplementary material for: GRIN2A null variants confer a high risk for early-onset schizophrenia and other mental disorders and potentially enable precision therapy
Source: Mol Psychiatry. 2025 Oct 14;31(1):374–82. doi: 10.1038/s41380-025-03279-4 (PMC12700805; doi:10.1038/s41380-025-03279-4)
Supplement: Supplementary file 1 — Supplementary Information [file 41380_2025_3279_MOESM1_ESM.pdf]

Supplementary information

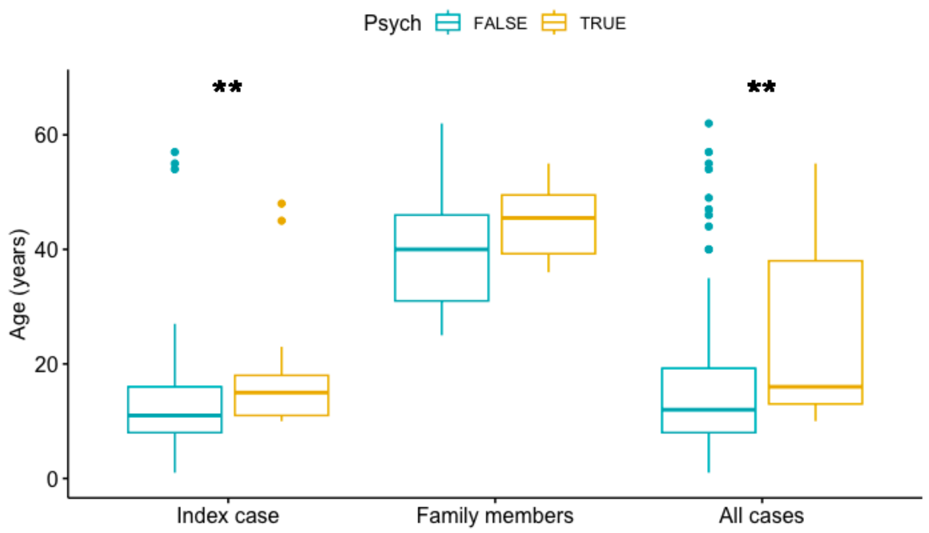

Figure S1 – Age comparison by groups and mental disorder presence

The boxplot illustrates the age distribution across different subgroups of the GRIN2A study cohort, segmented by the presence of mental disorders ('Psych'). The significance levels (Wilcoxon test) of the age distribution comparison between individuals with and without mental disorders are denoted as follows: * ($p < 0.05$), ** ($p < 0.01$), and *** ($p < 0.001$).

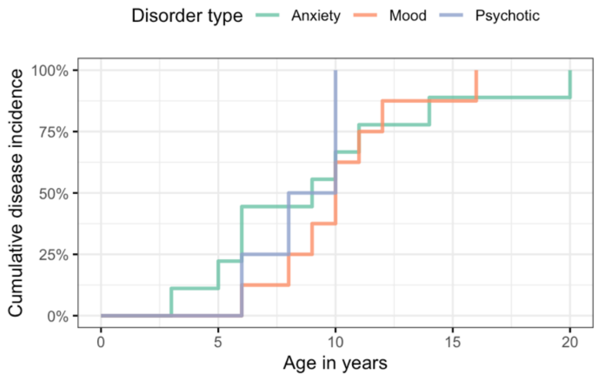

Figure S2 – Cumulative disease incidence by mental disorder, in carriers of *GRIN2A*$_{null}$ variants

Cumulative incidence curves of anxiety disorders, mood disorders, and psychotic disorders, in carriers of *GRIN2A*$_{null}$.

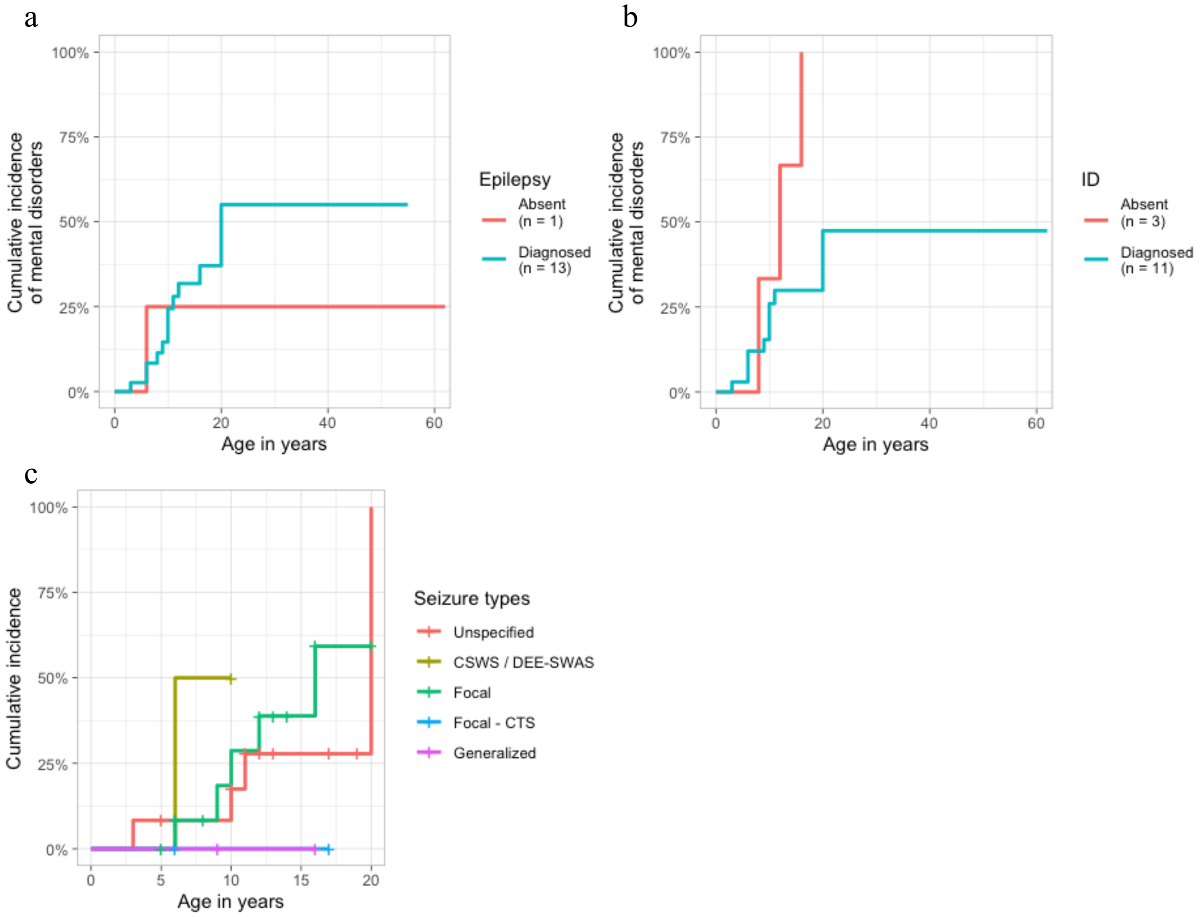

Figure S3 – Cumulative incidence of mental disorders for *GRIN2A*$_{\text{null}}$ carriers, by presence of epilepsy, intellectual disability (ID), seizure type.

a-b) The blue line represents individuals with a diagnosis of epilepsy (a) or of intellectual disability (b), the red line represents individuals without the corresponding diagnoses. N is the number of cases per each category. **c)** Available seizure types are continuous spikes and waves during sleep (CSWS/DEE-SWAS), focal, focal – CTS (centrotemporal spikes), generalized, and unspecific. (see Supplementary Table 4 for exact endpoint definitions)
